# Acoustic system for the estimation of the temporary blood chamber volume of the POLVAD heart supporting prosthesis

**DOI:** 10.1186/1475-925X-11-72

**Published:** 2012-09-21

**Authors:** Grzegorz Konieczny, Zbigniew Opilski, Tadeusz Pustelny, Maciej Gawlikowski

**Affiliations:** 1 Department of Optoelectronics, Silesian University of Technology, 2A Akademicka Str, 44-100, Gliwice, Poland; 2 Foundation of Cardiac Surgery Development, Zabrze, Poland

**Keywords:** Heart Assist Device, The heart prosthesis, Blood volume measurements, The POLVAD

## Abstract

**Background:**

The paper presents a newly researched acoustic system for blood volume measurements for the developed family of Polish ventricular assist devices. The pneumatic heart-supporting devices are still the preferred solution in some cases, and monitoring of their operation, especially the temporary blood volume, is yet to be solved.

**Methods:**

The prototype of the POLVAD-EXT prosthesis developed by the Foundation of Cardiac Surgery Development, Zabrze, Poland, is equipped with the newly researched acoustic blood volume measurement system based on the principle of Helmholtz’s acoustic resonance. The results of static volume measurements acquired using the acoustic sensor were verified by measuring the volume of the liquid filling the prosthesis. Dynamic measurements were conducted on the hybrid model of the human cardiovascular system at the Foundation, with the Transonic T410 (11PLX transducer - 5% uncertainty) ultrasound flow rate sensor, used as the reference.

**Results:**

The statistical analysis of a series of static tests have proved that the sensor solution provides blood volume measurement results with uncertainties (understood as a standard mean deviation) of less than 10%. Dynamic tests show a high correlation between the results of the acoustic system and those obtained by flow rate measurements using an ultrasound transit time type sensor.

**Conclusions:**

The results show that noninvasive, online temporary blood volume measurements in the POLVAD-EXT prosthesis, making use of the newly developed acoustic system, provides accurate static and dynamic measurements results. Conducted research provides the preliminary view on the possibility of reducing the additional sensor chamber volume in future.

## Background

The ultimate goal of most cardiac development programs all over the world is an artificial heart prosthesis, but long term biocompatibility issues, as well as the lack of long-life batteries complicate the achievement of a satisfactory solution. Currently most of those programs are in the stage of developing artificial heart-supporting devices which assist the heart in pumping the blood and in many cases allow a partial or entire recovery of the heart muscle [1,2], averting the need of heart transplant (bridge to transplant). Two main types of them are available on the market – non-pulsatile and pulsatile ones. Nowadays in most cases the implanted heart supporting devices are of a non-pulsatile type.

In the opinion of a heart transplant specialists there is a large group of patients suffering from a heart failure in whom the pulsatile heart-supporting devices are recommended choice. There are cases of patients, especially with coagulation problems, in whom external pulsatile devices provide better control of the supporting process. The pulsatile devices are a better choice in treatment of children [3]. It makes researches of pulsatile devices still an actual topic of a great medical importance.

In Poland the special National Program [see Additional file 1 for details], coordinated by the Foundation of Cardiac Surgery Development, Zabrze, was initiated for the purpose of developing a family of heart supporting prostheses including extracorporeal and fully implantable solutions. The National Scientific Polish Artificial Heart Program focuses on the development of a family of heart prostheses and their automation [4-7]. The Department of Optoelectronics at the Silesian University of Technology in Gliwice has been developing solutions for monitoring the POLVAD (Polish Ventricular Assist Device) prosthesis for this program since the time it has been started [8-11]. The POLVAD prosthesis is an extracorporeal pneumatic heart assist device. Its construction is similar to that of other heart assist devices of such a type (Thoratec VAD’s [12], AB5000 [2,13], Berlin Heart Excor [14], etc.), therefore the solutions developed for the Polish prosthesis can be implemented in similar pneumatic heart assist devices solutions (e.g. in a heart prosthesis Berlin Heart Excor VAD [14]). The Program includes a few tasks, among which we can distinguish the development of the prosthesis, driving systems of this prosthesis and sensors for its monitoring. A spectrum of heart prostheses models including fully implantable internal solutions will be the final result of the Program. Aiming at reaching a final solution a new heart supporting device model must be developed. Such a device will be based on the POLVAD device, modified in such a way that it may be implanted into a patient’s body. This condition requires changes in the construction of heart supporting prostheses, solving the biocompatibility issues and developing the monitoring system. One of the main issues is the automation of the heart supporting process that should be adjusted to the hemodynamic state of the supported patient. The automation system should be able to control the stroke volume and change it if needed. In order to achieve it, the feedback signal should be introduced. The proposed acoustic blood volume measurement system provides the solution for estimating temporary blood volume measurements by determining the actual volume of the air part, which is easier to analyze than the signal from sensors of the blood flow rate.

### The state of art

So far monitoring the blood pumping process of the POLVAD prosthesis has not been solved. As the POLVAD is made of semitransparent polymer materials, a monitoring system is not crucial, when the prosthesis is used extracorporeally. The medical staff can inspect its state at any point, and decide whether the heart supporting process is optimal. An additional information about the pumping process may be obtained by analyzing the pressure wave in the air duct between the POLPDU (Polish Pneumatic Driving Unit) and the POLVAD prosthesis. The operating conditions of heart supporting devices depend on cardiovascular impedances, which vary in time. The same driving pressures (SDP - systolic drive pressure and DDP – diastolic drive pressure) can induce different blood stroke volumes. Information about the air pressure in the pneumatic part can be used for the automation of the heart support, providing however only partial information about the state of the prosthesis. The blood volume should be measured along with the air and blood pressure and blood flow rate in order to provide a full information about the heart support process. This will allow to locate the heart supporting devices inside the human body, thus making the supporting process more friendly for the patient and increasing his safety and mobility.

**Figure 1:**
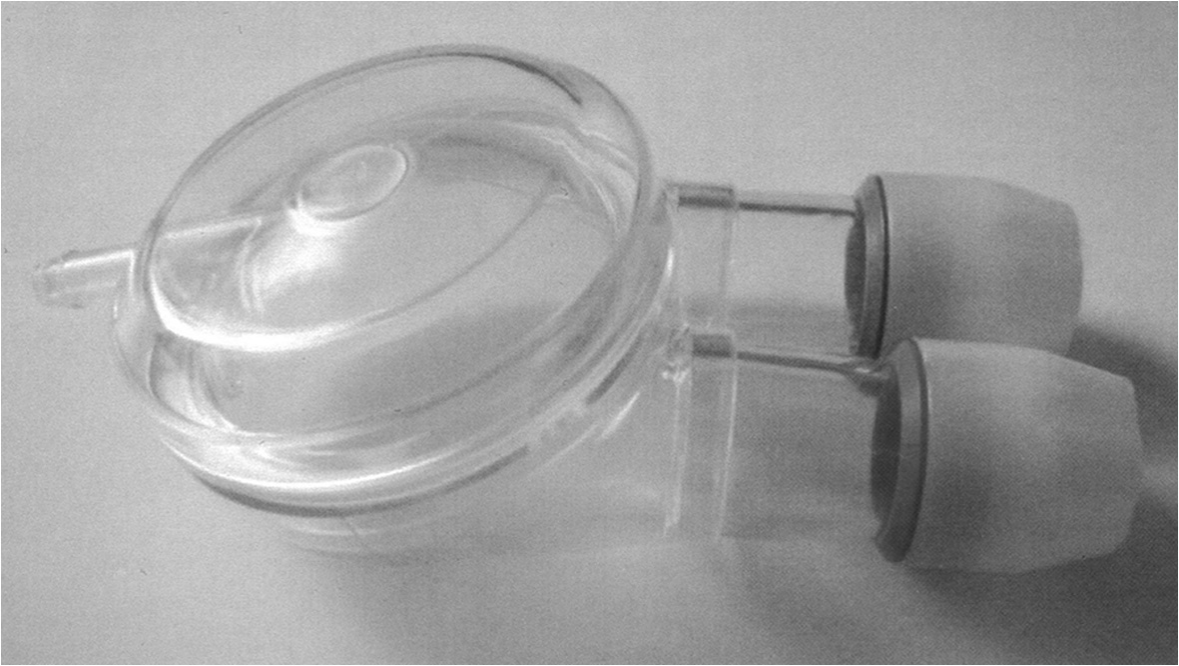
Polish heart supporting device prototype – POLVAD-EXT.

A pneumatic type prosthesis consists of the blood chamber, the air chamber and the membrane separating both chambers. There are two valves – one at the inlet and one at the outlet connector of the prosthesis, used to adjust the direction of the blood flow. Blood is pushed by the membrane, which is put into motion by the air pressure induced by means of the POLPDU. In the new POLVAD-EXT prosthesis (Figure 1) there is an additional volume above the pneumatic chamber, designed for housing of the electronics of monitoring systems. The maximum stroke volume of the POLVAD device usually amounts to 80 ml.

The measurements of the blood volume are essential to estimate the parameters of the blood flow (as the current stroke volumes) in the POLVAD prosthesis. The data from the sensor can then be used to automatize the driving process of the heart prosthesis.

There were approaches to the blood volume measurement problem in the POLVAD prosthesis in the past, none of them met the requirements to be incorporated into the final project of the POLVAD:

– inter-valve impedance measurements (high risk of blood clotting) [5],

– capacitance measurements (very sensitive to e-m noise) [5],

– acoustic white noise based method (complicated signal analysis, short life of the sensors exposed one-side to rapid pressure changes) [5],

– real time measurement of the casing properties of the prosthesis with the use of acceleration sensors (complicated and time consuming signal analysis) [6]

– optical amplitude sensor for the detection of the position of the membrane,

– image recognition of the membrane shape (requiring a small size, high speed camera and high computing power for real time pattern recognition) [7],

The mentioned methods of measurements involved errors (uncertainties) exceeding 10%.

Ultrasound blood flow sensors can be more accurate (5%) [15,16], but they require temporal scaling by a controlled full/empty cycle, and secondly - they permit blood flow measurements, but not directly the volume of blood in the prosthesis. The same problems occur when using air flow and pressure measurements for blood volume estimation in pneumatic type heart assist devices [17]. The history of the blood flow is necessary to estimate the temporary volume.

According to the authors knowledge, based on an extensive literature research, there is no solution allowing for the online temporary blood volume measurements, without the need of the measurements history, for pneumatic heart assist devices. The existing solutions allow only for boundary membrane positions detection (ie. the optical sensor in IVAD prosthesis from Thoratec Corporation [18]).

### Helmholtz’s resonator theory

**Figure 2:**
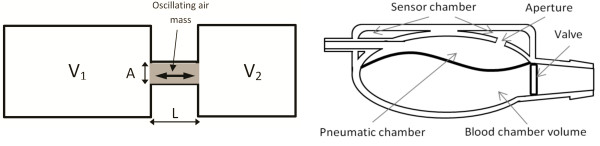
Helmholtz’s resonator theory and practical realization in POLVAD-EXT.

The newly developed method is based on Helmholtz’s acoustic resonance theory. The Helmholtz resonator is basically understood as a closed volume with an aperture, filled with gas. In most cases instead of an aperture there is a cylindrical neck (Figure 2a). It adds an additional dimension of “length of the neck” into the equations, which describe this resonator. The air “trapped” inside the neck vibrates with a frequency depending on the dimensions of the resonator [19]. The vibrations result from the springiness of the air inside the neck of the resonator.

If resonator’s volume and the volume outside the resonator are comparable in size, the system is treated as two resonators, sharing a common neck (Figure 2a). In that case both volumes ought to be considered [9]. In Figure 2b the application of the theory in the POLVAD-EXT prosthesis is presented.

(1) $$f≅\frac{c}{2\pi }\sqrt{\frac{A}{L}\left(\frac{1}{V_{1}}+\frac{1}{V_{2}}\right)}$$

Equation (1) shows, that the frequency of oscillation of the gas mass in the neck depends on: the volumes of the connected resonators (V_1_ and V_2_), the area (A) and the length (L) of the neck. This equation shows that it is possible to estimate the volume of one chamber, knowing the resonant frequency and the volume of the other one. (The application of the method of measuring the temporary blood chamber volume based on the Helmholtz resonator has been patented [11]).

### Researches of the prototype

Researches concerning the blood volume measurement system revealed the possibility of incorporating the acoustic system in an additional chamber attached above the POLVAD-MEV (clinically used extracorporeal prosthesis) pneumatic chamber. Tests provided promising results [10].

**Figure 3:**
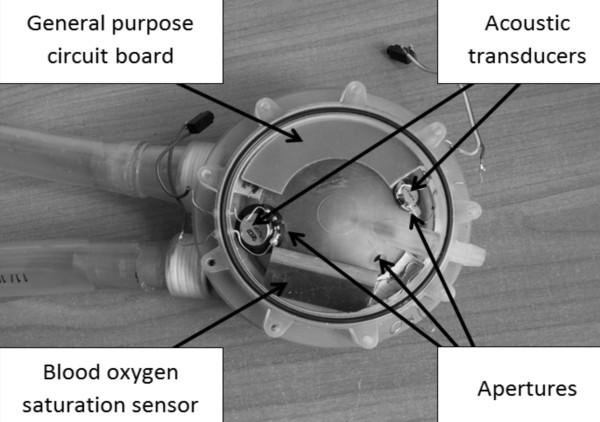
Schematic of the POLVAD-EXT prototype with the acoustic sensor prototype.

The decision was made to construct a prototype device. For this reason a new POLVAD-EXT prosthesis (the new model developed in the frames of the Polish Artificial Heart Program) with an additional chamber was designed (Figure 3). The prototype was manufactured in a rapid prototyping process.

Originally, the additional chamber was envisioned by the designers of the prosthesis as housing for the electronics of all sensors incorporated into the prosthesis. Compared to the previous model used for acoustic sensor tests [10], the additional chamber is reduced in size (35 ml) to form an integral part of the new prototype of the prosthesis. The constructed acoustic sensor uses modified additional space, without increasing its volume. In the POLVAD-EXT prototype the pneumatic part is closed by an additional cap, which restricts the space above the prosthesis; its construction allows to locate inside such a chamber both the acoustic transducers (transmitter and the receiver) and some electronic elements of other sensors. Three apertures were made between the air and the sensor chamber (Figure 3), providing the required modification of the frequency characteristics.

In order to test how the acoustic sensor behaves with additional circuit boards situated inside the sensor chamber, some additional elements were put in. One element substituted the general purpose circuit board; the second one was shaped like the blood saturation sensor circuit board (which will be used in the proposed form in the POLVAD-EXT).

### Electronic circuit

The system for acoustic blood volume measurements consists of sensor elements (a miniature speaker as a microphone and a speaker) and an electronic circuit. The sensor elements with the pneumatic and the sensor chambers form an acoustic part, and the electronic circuit realizes the positive feedback between the microphone and the speaker (acoustic signals are emitted to the sensor chamber by means of the speaker and are detected by the microphone).

The frequency is determined by means of a microcontroller, which counts time of five consecutive periods of the measurement signal and uses it for estimating the average signal frequency.

The result of frequency measurement is converted into the blood volume basing on the static characteristics determined during the static scaling. The results are displayed on the computer screen with a special application written in the LabView™ environment.

The development and researches concerning measurements of the blood volume in the POLVAD prosthesis can be divided into three main stages:

– preliminary tests of the Helmholtz resonator used in the POLVAD-MEV prosthesis [9],

– testing of the POLVAD-MEV prosthesis with an additional sensor chamber [10],

– development and testing of the prototype POLVAD-EXT prosthesis with the sensor chamber, dealt with in this paper.

All these three stages include modifications of the electronic circuit, in order to improve the stability of the sensor and the resulting measurements. While pumping the blood-like liquid, the POLVAD mechanical elements cause considerable acoustic noise. Since the proposed acoustic method works within the frequency range of 800-1400 Hz, the noise signal must be filtered. The most troublesome noise signal was caused by the mechanical blood valves with frequencies <500 Hz. In some cases the amplitude of the noise signal exceeded the useful measurement signal. It stopped the detection of the Helmholtz resonance; therefore methods of measurement signal filtration had to be developed. The filtration was accomplished by means of a low- pass electronic filter with the 1600 Hz cut off frequency. Three types of second-order electronic filters were tested: Chebyshev’s, Butterworth’s and Sallen-Key’s. The latter one proved to be most stable of all the tested types and suitable for the final model. Additionally the microphone was substituted by a small speaker realizing cut-off of <500 Hz frequencies, making the high pass filter redundant. After the described changes the measurement signal took a form of a single frequency sine wave at Helmholtz’s resonance frequency (in the range of 900–1600 Hz). After the presented modifications in the physical construction of the sensor (in the sensor chamber) and the electronic part, the acoustic sensor system with the computer analysis program permitted measurements of the blood chamber volume in real time with a satisfying accuracy.

## Methods

The acoustic system embedded in the prosthesis underwent static and dynamic tests. The static properties were measured at the Department of Optoelectronics and consisted of series of frequency measurements at certain volumes for assessment of the characteristics. The blood-like solution used during the measurements was a 60% water solution of glycerin mixed with benzoic aldehyde (capillary viscometer measurement result: 8.5 cP at 25°C). The actual volume of the liquid, filling the prosthesis, was determined by estimating the volume of liquid in the measuring cylinder, connected with the prosthesis. The volumes were measured every 4 ml in the range of 0-80 ml with 1 ml precision. The results of the static measurements were used to determine the frequency-volume characteristics used further in dynamic measurements.

The dynamic measurements were taken at the Foundation of Cardiac Surgery Development on the hybrid model [20,21] of a human vascular system. The prosthesis was driven by the POLPDU-402 driving unit, allowing to change the heart-supporting parameters up to SDP_max_ = +300 mmHg, DDP_max_ = −75 mmHg and AHR_max_ = 120BPM. The Transonic T410 ultrasound flowmeter with a PXL11 probe was used for flow rate measurements in the tube connected with an outlet drain. The ultrasound sensor used in the measurements was previously calibrated using the Fluxus ultrasound flowmeter (2% uncertainty). Several series of measurements were conducted at different SDP and DPP pressures and heart rate parameters.

Additionally mechanical clamps were used at the inlet and outlet tubes connected with the prosthesis in order to modify the preload and afterload parameters. During the measurements the results from both the acoustic system and the flow meter were collected. The acoustic system provided results of direct volume measurements. The results of the flow rate measurements were used to estimate the stroke volume.

The development of the implantable prosthesis requires in the future a limitation of the additional chamber volume. To examine the possibility of reducing the sensor chamber’s volume, a solid element with a volume of 12 ml was introduced in it. Preliminary acoustic sensor tests were performed with the volume of the sensor chamber limited to 23 ml (32% volume reduction).

## Results

**Figure 4:**
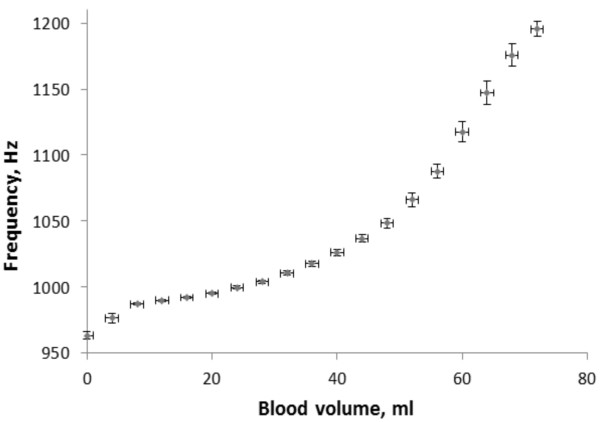
Static frequency vs. volume characteristics of prosthesis with the acoustic sensor.

The POLVAD-EXT prototype with the acoustic system for volume measurement was tested statically in a series of 25 measurements of frequency vs. volume characteristics (Figure 4).

**Figure 5:**
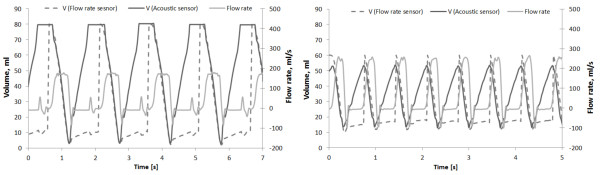
Exemplary results of the POLVAD prototype dynamic tests (AHR – average heart rate [1/min]).

The frequency-volume characteristic is monotonic (the frequency rises with the rising blood chamber volume) in the 0- ~ 72 ml range. Above 72 ml volume (below 8 ml pneumatic chamber volume) there is a decrease in frequency (with a rising blood volume) along with decreasing amplitude of the measurement signal. This can be used in measurements of the additional ~4 ml volume. Above 75 ml the Helmholtz resonance cannot be properly detected, thus the uncertainty of measurements of the full filling state is ~5 ml. The conducted statistical analysis of 25 static measurements showed that the uncertainty (understood as the standard mean deviation) of the volume measurements for the volumes <75 ml is less than 5%. The static measurements were used for scaling the sensor for dynamic measurements. The program was written in such a way, that when the amplitude of an acoustic signal is too low (>75 ml), the sensor system shows that the volume is 80 ml, and sends this result to the computer. This is the reason why there is no change in the volume measured by the acoustic sensor while the flow rate sensor shows the liquid flowing at volumes above ~75 ml (Figure 5a).

The graphs (Figure 5 a and b) show the results of acoustic blood volume measurements and flow rate measurements at the output cannula. The flow rate was used to estimate the output volume of the prosthesis. For a better comparison of volumes measured by means of both methods the maximum value of the volume obtained by the flow rate sensor is set to 80 ml (Figure 5a) and 60 ml (Figure 5b).

Exemplary graphs present measurements results of full filling/full ejecting (Figure 5a) and of partial filling/partial ejecting (Figure 5b) prosthesis work modes. The first mode is preferable, but the results show that the sensor can measure transitional blood chamber volumes.

**Figure 6:**
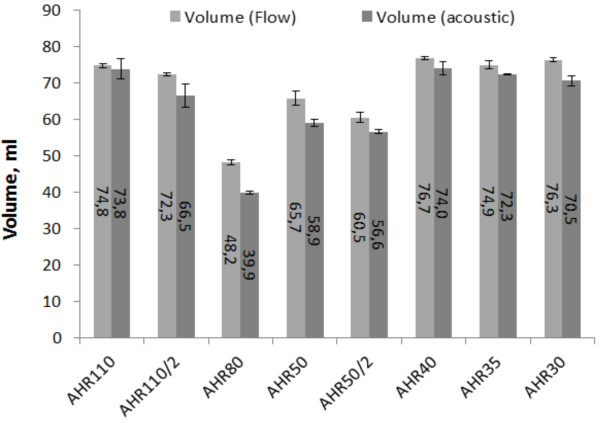
Results of the acoustic volume measurements compared with the volume obtained with the flow rate measurements.

Eight measurement series were carried out using different supporting parameters, forcing the prosthesis to work with different stroke volumes. The reference measurements were made with use of the ultrasound *Transonic* sensor of the transit time type. The average stroke volumes measured by means of the acoustic sensor and the volumes calculated basing on the results of the flow rate ultrasound sensor results are compared in Figure 6.

The results acquired by measurements with the use of both the acoustic and reference ultrasound flow rate measurement systems are similar. At AHR 80 the difference between the results is 8.2 ml, which is the maximum difference of all the measured series.

The flow meter should detect the flow of the blood-like liquid only when the POLVAD prosthesis works in the ejecting cycle (high flow meter readings at a falling edge of the blood volume curve). There is, however, an additional negative flow rate signal at the output of the cannula during the ejection and filling (Figure 5). These phenomena can be explained by the operation of the POLVAD valves. During the pumping cycles, when one valve is open, the other one is closed. Although the other valve should close almost instantly, there is some delay during which it remains half-open. In this time the like-blood liquid can be driven back to the blood chamber, which results in a small but measurable negative blood flow. The leakage also occurs when the valves are completely closed. The tested acoustic measurement system worked correctly in the whole range of admissible supporting pulsations and pressures of the driven air. The tests showed that the acoustic sensor permits proper measurements even when the POLVAD works above the safe heart support parameters (SDP > 180 mmHg, working in the partial filling/partial emptying mode).

**Figure 7:**
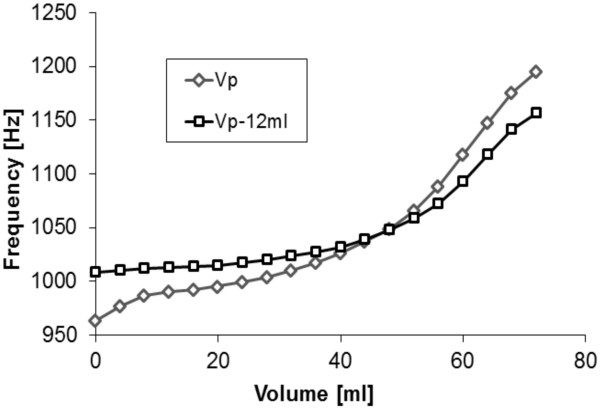
Comparison of the static frequency characteristics of the prototype with and without volume reducing elements inside the sensor chamber.

The test concerning the behavior of the acoustic sensor was performed in the case of a limited volume of the sensor chamber. The static characteristic was determined. Figure 7 illustrates both static characteristics for the prototype with (Vp-12 ml) and without (Vp) the additional volume limiting element.

As expected, the volume reduction caused a change in the frequency vs. volume characteristics of the sensor. The frequency difference between the boundary volumes was reduced from ~300 Hz (for the regular sensor chamber) to ~160 Hz (for the chamber with the volume limiting element.). It shows that the reduction of the additional volume decreases the sensor sensitivity.

Test results show that a further reduction of the chamber volume is possible in restricted range, thus the total volume of the POLVAD prosthesis can still be limited in the future. The compromise should be met though between the size of the additional chamber and the volume used by the electronics. Decrease in the volume of the electronics would effectively provide additional volume for the acoustic sensor and should allow the future miniaturization of the sensor chamber size without the decrease of the sensitivity of the acoustic sensors.

Further examinations with a limited sensor chamber volume are required to support fully this claim and to estimate the uncertainty of measurements depending both on the additional volume and on the shape of the chamber.

## Discussion

Solutions concerning ultrasound flow rate sensors permit to estimate the volume of blood with a relatively high accuracy. Using e.g. the Transonic 11PLX ultrasound sensor one can obtain values of flows with an accuracy of ±5%. The main disadvantage of the ultrasound sensors is their size (hindering the possibility of use in the implantable version of the pneumatic, pulsatile prostheses) and complicated signal analysis, which requires sophisticated hardware drivers. It limits the use of these methods to clinical cases. This type of sensors also requires using the history of the results of a blood volume from previous cycles. Recent calibration, by means of an independent method of entire empty (entire filling) state detection is required.

The proposed acoustic sensor permits direct blood volume measurements by estimating the position of the membrane (pneumatic chamber volume measurements). The idea of the Helmholtz’s resonator proposed in this paper (using the pneumatic chamber and additional sensor chamber on top of the pneumatic chamber) seems to be the most reasonable construction. Other approaches (using air drain connecting the prosthesis with the POLPDU unit) would cause a resonance with very low frequencies (<1 Hz) making it difficult to analyze the blood volume with the required accuracy.

The dependence of the measured volume vs. frequency characteristic on changes of the air pressure in the pneumatic part of the prosthesis is eliminated by its opposite dependence on the changes of the air density (the influence of air pressure and air density change on sound velocity is cancelled out). The resonant frequency (and thus also the sensing system) depends on the temperature (in Kelvin scale), because the velocity of sound also depends on temperature. Thus in the final solution the numerical correction considering changes of temperature of the air might be advisable. In the actual form of the POLVAD prototype the air flow circuit between the prosthesis and the driving unit is closed, so the effect of temperature is at its minimum. No preload and afterload influence on the volume measurements was observed, since these parameters do not affect the properties of the pneumatic chamber. The final solution will require high quality acoustic transducers. Acoustic transducers used in the construction of the prototype were not destroyed by the rapid pressure changes occurring in the pneumatic part of the prosthesis after many months of investigations of the sensor system.

Apertures in the intermediate backplane of the prosthesis are 5 mm in diameter, and are not a risk to the membrane if driving the prosthesis (pneumatic part driving pressures) is correlated with the measurements of the volume.

The system will be used in an extracorporeal version of the prosthesis in the first stage of testing on animal body. Furthermore, in new models of the POLVAD prosthesis with different volumes and shapes their acoustic characteristics will differ, and a new calibration will be necessary. The reduction of the additional volume will be necessary for the implantable version. Preliminary tests show that the reduction of volume of the additional chamber narrows the f(V) frequency range thus additional researches are required aiming at enhancing the characteristics of the sensor in the limited sensor chamber volume situation as well as by using electronic elements in the form of specialized integrated electronic elements with very small volumes.

Further measurements, using the real blood environment are indispensable for a final evaluation of the sensor. The presented system will be tested in near future on animals.

## Conclusions

The problem of the estimation of the temporary blood volume in the pulsatile type heart prosthesis has been present for over 30 years [22] and is yet to be solved. The previous approaches to this topic were mainly based on two ideas: the measurements of the air mass and pressure in the pneumatic part of the prosthesis and the ultrasound measurements of the blood flow. Both of these methods require periodic calibration cycles (the additional method is needed for accurate estimation of the full filling/full ejection state of the prosthesis). The estimation of the full filling/full ejection state basing only on the measurement of the air parameters in the air chamber is difficult since the cardiovascular impedances are different for each patient and additionally vary in time; this causes the error of the calibration of the “zero volume” which influences the overall accuracy of flow rate based temporary volume measurements.

The acoustic sensor provides direct measurements of the volume of blood inside the POLVAD prosthesis, because the actual blood volume is determined basing on the actual volume of the air part (the measurements results do not depend practically on the patients impedances). Very essential is that the sensor does not contact the blood and is absolutely medically safe. The elaborated system provides results without the necessity of knowing the history of measurements (in order to determine the actual volume of the blood chamber the result of a previous measurement is not required). The proposed method is much less complicated than the concurrent solutions and does not require a sophisticated analysis of the results of measurements.

Presently, it provides the best accuracy of direct measurements (among sensors incorporated into the chamber construction) of the temporary blood volumes in the pulsatile type heart-supporting devices.

## Competing interests

The authors declare that they have no competing interests.

## Authors’ contributions

“GK participated in all of researches, participated in development of the measurement method, carried out the data analysis and drafted the manuscript.ZO conceived of the study, participated in all of researches and development of the method, carried out data acquisition and carried out data analysis. TP participated in the design and coordination of the study and revised the manuscript. MG participated in the dynamic measurements on the measurement stand at the Foundation of Cardiac Surgery Development. All authors read and approved the final manuscript.

## Supplementary Material

Additional file 1 **Appendix 1.** Information on Polish Artificial Heart Program. Click here for file
