# Supplementary material for: Acoustic system for the estimation of the temporary blood chamber volume of the POLVAD heart supporting prosthesis
Source: Biomed Eng Online. 2012 Sep 21;11:72. doi: 10.1186/1475-925X-11-72 (PMC3502557; doi:10.1186/1475-925X-11-72)
Supplement: Additional file 1 — Appendix 1. Information on Polish Artificial Heart Program. [file 1475-925X-11-72-S1.docx]

**Appendix1 - Polish Artificial Heart Program**

In 1994 the Foundation of Cardiac Surgery Development (Zabrze, Poland) proposed a system for supporting the human cardiovascular system named POLCAS (Polish Cardiac Assist System) [a]. Two years later the system has been introduced to clinical use [b, c]. From 1999 to February 2012 the POLCAS system was clinically used in over 250 patients: with III and IV NYHA class myocardial insufficiency, with dilated cardiomyopathy of heart attack or inflammation etiology [d, e]. In 80% of cases of the mechanical heart support was used as a „bridge to transplant”, in 19.9% as „bridge to recovery”. The rest of mechanical heart support cases were used in patients qualified for a heart transplantation surgeries [f, g, h]. In the mentioned cases the univentricular and biventricular heart support was realized with use of a total of 590 extracorporeal, pneumatic, pulsatile ventricular assist devices (POLVAD-MEV). The POLCAS system was introduced to six clinics in Poland. Currently in four of them the system is being actively used.

The many years’ experience and a unique knowledge gained during the clinical use of the POLCAS system were utilized for the newly started researches aiming at the improvement of the mechanical heart supporting system used so far. On the 6th of March 2007 [i] the long-term Polish Artificial Heart Program was founded . The main goals of the program include:

- development of a family of polish heart prostheses,
- improvement in clinical usage of polish heart prostheses in treating patients with a critical myocardial insufficiency,
- foundation of a highly-specialized science-technological platform aiming at conducting comprehensive research and development in terms of heart prostheses.

The family of developed heart prosthesis includes: pulsatile extracorporeal heart prosthesis (for short-term and long-term heart supporting periods), pulsatile, partially-implantable prosthesis (for long-term heart supporting periods), rotary blood pump (for short-term heart supporting in acute or critical myocardial insufficiency, myocardial infraction or postoperative insufficiency) and a totally implantable prosthesis (for long-term heart supporting) [j, k, l].

The Polish Artificial Heart Program consists of five enterprises, grouping a total of 29 of research and implementation tasks. Individual tasks cover the disciplines fundamental for the construction of heart prostheses and their clinical implementation [k]:

- Enterprise P01: "Development of technologies of material engineering, surface engineering and bioengineering for use in the heart prostheses”
- Enterprise P02: "Development of metrological, informatic, and teleinformatic technologies for use with the heart prostheses”
- Enterprise P03: "Development of a construction of clinical heart supporting systems”
- Enterprise P04: "Clinical implementation of methods for treatment of a myocardial insufficiency using polish heart prostheses”
- Enterprise P05: " Foundation of a highly-specialized science-technological platform aiming at conducting comprehensive research and development in terms of heart prostheses ".

Contractors realizing individual tasks are chosen by open competition. The Polish Artificial Heart Program is being realized by: Polish Minister of Health, Polish Minister of Science and Higher Education and the Polish National Centre for Research and Development. The Foundation of Cardiac Surgery Development [m] is coordinating the Program.

**References**

[a] Nawrat Z.: Zbigniew Religa: An unfinished story. The International Journal of Artificial Organs / Vol. 32 / no. 6, 2009 / pp. 315-317

[b] Sitkowska-Rysiak E., Partyka T., Gutowska B., Banas S. et al.: Our experiences with the POLVAD-MEV ventricular assist device. Anestezjologia Intensywna Terapia 2/2002, p. 114-116

[c] Pacholewicz J., Kucewicz E., Zakliczyński M., Niklewski T., Kubacki K., Kustosz R., Zembala M.: Heart muscle regeneration after long-term treatment using a POLVAD mechanical ventricular assist device – case study. Kardiochirurgia i Torakochirurgia Polska 2008, 5(3). (In Polish)

[d] Litwiński P., Religa G., Pastuszek M., Parulski A., Religa Z. et al.: The mechanical blood circulation using POLVAD ventricular assist devices in treatment of cardiogenic shock caused by a heart muscle inflamation. Kardiochir Torakochir Polska 2005. 2(4). (In Polish)

[e] Dobrowolski P., Szymański P., Religa G., Różański J.: Echocardiographic examination after implantation of artificial heart prostheses. Kardiologia Polska, 67, 2009 (In Polish)

[f] Jarmoszewicz K., Siondalski P., Pawlaczyk R., Brzeziński M., Rogowski J.: Treatment of an acute heart failure. Clinical cases. Kardiochirurgia i Torakochirurgia Polska 2008; 5 (1): 52–55 (In Polish)

[g] Pacholewicz J., Zakliczyński M., Barańska-Kosakowska A., Religa G., Siondalski P., Kucewicz E., Nadziakiewicz P., Zembala M.: Efficacy of treatment with pulsatile pump as a bridge to transplantation in patients with a congestive heart failure - polish experiences with POLVAD. Heart Lung Transplant.2009; Vol.28, No.2S, p.S98, [93]

[h] Pacholewicz J., Hrapkowicz T., Chodór B., Kubacki K. et. al: Good long-term result of biventricular assisted circulation using POLCAS-RELIGA system in 16-year old boy with a irreversible heart failure - a case report. Kardiochir.Torakochir.Pol.2009; T.6, nr 4, s.377-383

[i] online: http://www.mz.gov.pl/wwwmz/index?mr=m101&ms=&ml=pl&mi=126&mx=0&mt=&my=152&ma=09363

[j] online: http://pwpss.pl/

[k] El Fray M., Czugala M.: Polish artificial heart program, WIREs Nanomed Nanobiotechnol 2011. doi: 10.1002/wnan.175

[l] Szymański P., Religa G., Klisiewicz A., Barańska K, Hoffman P.:  Diagnosis of biventricular assist device inflow cannula obstruction. Echocardiography, Volume 24, Issue 4, April 2007, Pages 420-424

[m] online: http://www.frk.pl
